# Effects of sitagliptin beyond glycemic control: focus on quality of life

**DOI:** 10.1186/1475-2840-12-35

**Published:** 2013-02-21

**Authors:** Yoshiko Sakamoto, Jun-ichi Oyama, Hideo Ikeda, Shigetaka Kuroki, Shigeki Gondo, Taketo Iwamoto, Yasufumi Uchida, Kazuhisa Kodama, Atsushi Hiwatashi, Mitsuhiro Shimomura, Isao Taguchi, Teruo Inoue, Koichi Node

**Affiliations:** 1 Department of Cardiovascular Medicine, Saga University, 5-1-1 Nabeshima, Saga city, Saga, 849-8501, Japan; 2 Saga Medical Association, Saga, Japan; 3 Department of Cardiovascular Medicine, Dokkyo Medical University, Mibu, Tochigi, Japan

**Keywords:** DPP-4 inhibitor, Diabetes type 2, HbA1c, Blood pressure, Metabolism

## Abstract

**Background:**

Recently, incretin hormones, including glucagon-like peptide-1 (GLP-1) analogue and dipeptidyl peptidase-4 (DPP-4) inhibitor, have been found to regulate glucose metabolism. The aim of this study was to elucidate the efficacy and safety of the clinical usage of DPP-4 inhibitors in Japan.

**Methods:**

This study was designed as a prospective, open-label, multi-center trial. Patients with diabetes mellitus type 2 (T2DM) with poor glycemic profiles (HbA1c ≥ 6.2%) in spite of receiving a medical diet, therapeutic exercise, and/or medications were eligible for this study. The participants received 50 to 100 mg of the DPP-4 inhibitor sitagliptin once daily for 12 months.

**Results:**

One hundred and eighty-eight subjects were enrolled. After 12 months of sitagliptin treatment, HbA1c levels decreased (7.65% ± 1.32% to 7.05% ± 1.10%, p < 0.001) as well as fasting plasma glucose (FPG) (145 ± 52 mg/dl to 129 ± 43 mg/dl, p = 0.005). The rate of glycemic control achieved (in accordance with the guidelines of the Japanese Diabetes Society) significantly increased. Blood pressure and serum levels of triglycerides and total cholesterol decreased significantly. Furthermore, the Pittsburgh Sleep Quality Index (PSQI) and Diabetes Symptomatic Scores improved significantly. Adverse events such as hypoglycemia and loss of consciousness occurred in twenty three subjects (11%).

**Conclusions:**

These results suggest that the actions of DPP-4 inhibitors improve not only glycemic control, but also blood pressure, lipid profiles, and quality of life (QOL). Sitagliptin is a sound agent for use in the comprehensive treatment of patients with T2DM.

## Introduction

In Japan, the Ministry of Health, Labour and Welfare published a report on health and nourishment in 2007 [1] that estimated that 22.1 million people have strongly suspected diabetes mellitus (DM) (HbA1c (NGSP) ≥ 6.5%) or potential DM (6.0% ≤ HbA1c (NGSP) < 6.5%). This rate has increased 1.3 times compared to that observed in the former decade, and an upward trend continues to be maintained. Additionally, the rate of diabetic treatment has increased compared to that of 10 years ago. However, it has been reported that 36.5% of affected patients have not received diabetic treatment because conventional anti-diabetic drugs are inconvenient to use and exhibit inadequate efficacy, a short duration of activity, and side effects such as hypoglycemia, weight gain, and digestive symptoms. Therefore, these drugs are associated with problems regarding safety and tolerability. In 2006, the US Food and Drug Administration approved the dipeptidyl peptidase-4 (DPP-4) inhibitor sitagliptin. DPP-4 inhibitors are a new class of anti-diabetic drugs that exhibit different mechanisms of action from conventional anti-diabetic drugs.

Sitagliptin binds to DPP-4 and prevents the breakdown of glucagon-like peptide-1 (GLP-1) and glucose-dependent insulinotropic polypeptide (GIP) [2]. Both GLP-1 and GIP are types of incretin hormones released by the intestines that stimulate insulin secretion from β cells [3] and suppress glucagon secretion [4]. GLP-1 and GIP are rapidly broken down by DPP-4 [5]. Incretin hormones depend on the level of blood glucose to stimulate insulin. DPP-4 inhibitors are associated with a lower incidence of hypoglycemia than conventional hypoglycemic drugs.

This study is a single-arm, prospective, multi-center trial conducted to evaluate the efficacy and safety of the DPP-4 inhibitor sitagliptin in clinical use. In this trial, we particularly focused on the effects of sitagliptin on quality of life (QOL).

## Methods

### Study design and protocol

The Institutional Review Board of Human Research at Saga University approved this study and informed consent was obtained from all participants. Patients with T2DM (age ≥ 20 years) with poor glycemic control profiles [HbA1c ≥ 6.2%, as evaluated according to the National Glycohemoglobin Standardization Program (NGSP)] in spite of receiving a medical diet, therapeutic exercise, and/or conventional anti-diabetic medications were recruited. The exclusion criteria were treatment with insulin, a history of severe diabetic ketoacidosis or coma, severe infection, perioperative state, severe trauma, pregnancy, breast-feeding, renal dysfunction (creatinine clearance < 30 ml/min or serum creatinine: male: ≥ 1.5 mg/dl, female: ≥ 1.3 mg/dl), a history of experiencing side effects to sitagliptin or other unsuitableness. For the participants, sitagliptin was given as either a new prescription, as an additional prescription to other conventional anti-diabetic agents, or replaced other anti-diabetic drugs.

The subjects received 50 mg sitagliptin, once a day for the first 3 months. After 3 months, the dose of sitagliptin was changed to between (and including) 25 mg/day and 100 mg/day, and other oral hypoglycemic drugs were added according to the discretion of each physician. The observation period was 12 months.

### Clinical measurements

After 12 months of treatment with sitagliptin, changes in HbA1c, fasting plasma glucose (FPG), blood pressure, body weight (BW), body mass index (BMI), total cholesterol (TC), LDL cholesterol (LDL-C), HDL cholesterol (HDL-C), triglycerides (TG), 1.5-anhydro-D-glucitol (1.5-AG), microalbuminuria, and homeostasis model assessment analyses of beta cell function (HOMA-β) and insulin resistance (HOMA-IR) were assessed. We also assessed changes in the subjects’ quality of life (QOL) using the Euro QOL (EQ)-5 Dimensions (EQ-5D), the EQ Visual Analogue Scale (EQ-VAS), the Pittsburgh Sleep Quality Index (PSQI), and the Diabetes Symptomatic Score.

The EQ-5D is a generic instrument for measuring health-related QOL that has been developed and validated in a number of European countries [6,7]. The EQ-5D describes a patient’s health status according to five dimensions: mobility, self-care, usual activities, pain/discomfort and anxiety/depression. Each dimension has three levels that include no problems, some problems or severe problems. This yields 243 potential combinations of health states across the five dimensions. Dolan et al. [8] measured 42 of these health states in a representative sample of the United Kingdom general population using the Time Trade-Off method [9]. Based on these evaluations, the utility scores can be deduced by means of an additive function. The utility scores may vary between −0.59 (worst health) and 1.00 (perfect health). In addition to the five dimensions, the EuroQol consists of an EQ-VAS ranging from 0 (worst imaginable health state) to 100 (best imaginable health state) [10]. The PSQI is a self-administered questionnaire used to assess subjective sleep quality during the previous month [11]. The self-rated items of the PSQI generate seven component scores (range of subscale scores: 0 to 3) for sleep quality, sleep latency, sleep duration, habitual sleep efficiency, sleep disturbance, use of sleep medications, and daytime dysfunction. The sum of these seven component scores yields one global score of subjective sleep quality (range: 0 to 21), with higher scores representing poorer subjective sleep quality. The psychometric properties of the PSQI have been confirmed in previous studies [11,12]. We have used 5.5 points as a cut-off in the Japanese version of the PSQI global score [6]. The Diabetes Symptomatic Score is a method for assessing QOL that was originally developed for the S-DOG trial. This score is calculated as the sum of the scores, graded 1 to 5, for 10 diabetes-related symptoms (Table 1).

**Table 1:** Checklist of diabetes symptomatic score

	**Checklist**
1	Are you often thirsty?
2	Do you produce urine frequently?
3	Are you worried about urinary smell?
4	Do you feel numbness of your extremities?
5	Do you have edema in your legs?
6	Do you have cramps in your legs?
7	Are you insensitive to the pain of a small wound or burn?
8	Do you have a feeling of listlessness?
9	Do you feel lightheaded?
10	Is your vision blurry? Is your eyesight getting worse?

Diabetes Symptomatic Score is used to measure the grade of ten diabetic symptoms. Patients with a high score have worsening of diabetic symptoms.

None: 0 point, Rare: 1 point, Sometimes: 2 points, Frequent: 3 points, Always: 4 points. Maximum: 40 points.

### Statistics

Values are expressed as the mean ± SD. To compare changes in the values of HbA1c, FPG, BW, BMI, BP, lipids, 1.5AG, and HOMA from baseline to after 12 weeks of treatment, we used the paired *t*-test. To compare changes in the values of the EQ-5D, EQ-VAS, PSQI, and Diabetes Symptomatic Score, we used the Wilcoxon signed-rank test. Values of p < 0.05 were considered to be statistically significant.

## Results

### Baseline characteristics

**Figure 1:**
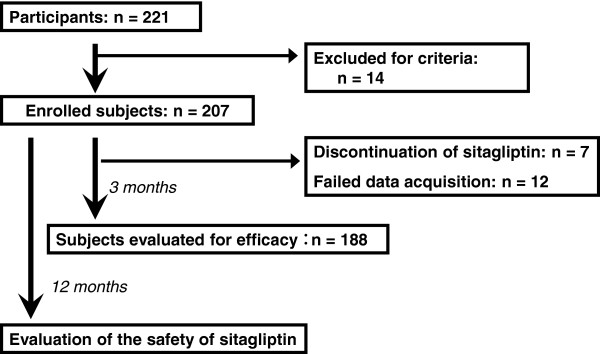
Study Enrollment.

A total of 221 patients agreed to participate in this study. Of the 221 patients, 14 were excluded due to protocol violation. Among the 207 enrolled subjects, seven were excluded due to discontinuing sitagliptin within the first 3 months, and 12 were excluded because data acquisition to evaluate the efficacy of the drug failed. Therefore, sitagliptin efficacy over 3 months was evaluated in 188 subjects as efficacy population. The safety of sitagliptin over 12 months was also evaluated in the 207 enrolled subjects as safety population (Figure 1).

Table 2 shows the clinical characteristics of the study subjects prior to the start of treatment with sitagliptin. The average age of the evaluated subjects was 66.9 years, 91 subjects (48%) were male, the mean duration of diabetes was 6.9 years and the mean HbA1c level was 7.65% at baseline.

**Table 2:** Baseline characteristics

	**Enrolled subjects**	**Evaluated subjects**
	**(n = 207)**	**(n = 188)**
Age (years)	66.5 ± 12.8	66.9 ± 12.6
Gender	Male: 50% (n = 103),	Male: 48% (n = 91),
	Female: 50% (n = 104)	Female: 52% (n = 97)
BMI	25.0 ± 4.4 kg/m^2^	25.0 ± 4.4 kg/m^2^
Waist circumference	89.4 ± 12.8 cm	89.0 ± 12.8 cm
Obesity (BMI > 25)	51%	50%
Duration of DM (years)	6.8 ± 6.5	6.9 ± 6.6
Smoking status	Smoker: 24%	Smoker: 23%
	Past smoker: 13%	Past smoker: 13%
	Never: 63%	Never: 63%
Alcohol consumption	Yes: 30%	Yes: 29%,
Complications	HT: 67%, DL: 55%, HUA: 7%,	HT: 65%, DL: 55%,
	Arrhythmia: 5%, CKD 43%	HUA: 6%, Arrhythmia: 5%,
		CKD 43%
Use of sitagliptin	New: 35%	New: 35%
	Added: 45%, Changed: 20%	Added 45%, Changed 20%
Combined drugs	SU: 49%, BG: 20%, TZD: 28%,	SU: 48%, BG: 22%,
	Glinide: 2%, α-GI: 7%	TZD: 28%, Glinide: 2%, α-GI: 7%

BMI, Body mass index; DM, diabetes mellitus; HT, hypertension; DL, dyslipidemia; HUA, hyperuricemia; CKD, chronic kidney disease; SU, sulfonylurea; BG, biguanide; TZD, thiazolidinedione; α-GI, α-glucosidase inhibitor.

### Effects of sitagliptin on glycemic control

**Figure 2:**
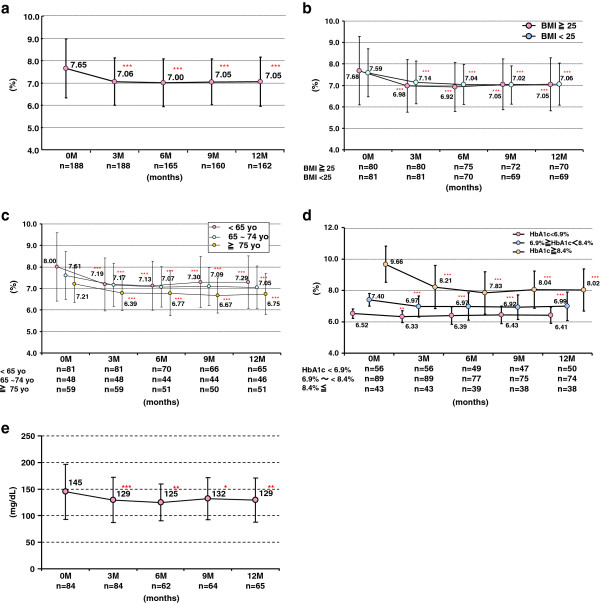
**Serial HbA1c changes in (a) all subjects, (b) BMI-based groups, (c) age-based groups, (d) HbA1c-based groups, and (e) fasting plasma glucose.** HbA1c, hemoglobin A1c; BMI, body mass index. *p < 0.05, **p < 0.01, ***p < 0.001, vs. baseline by paired t-test.

Overall, HbA1c levels decreased in all of the 188 evaluated subjects after 3 months (7.65% ± 1.32% to 7.06% ± 1.07%, p < 0.001) and 12 months (7.05% ± 1.10%, p < 0.001) of sitagliptin treatment (Figure 2a). The HbA1c decreases per subgroup are described here.

For the 66 subjects who received sitagliptin alone, the decreases were 7.44% ± 1.31% to 6.72% ± 0.82%, p < 0.001 at 3 months and 6.61% ± 0.82%, p < 0.001 at 12 months. Eighty-five subjects received sitagliptin along with other anti-diabetic agents, HbA1c level decreases were 7.86% ± 1.25% to 7.22% ± 1.18%, p < 0.001, 3 months and 7.32% ± 1.20%, p < 0.001, 12 months. In subjects with a BMI < 25 kg/m^2^ (n = 81) and those with a BMI ≥ 25 kg/m^2^ (n = 80), a decrease in HbA1c levels was observed after 12 months of sitagliptin treatment: 7.59% ± 1.16% to 7.06% ± 1.12%, p < 0.001 and 7.68% ± 1.47% to 7.05% ± 1.03%, p < 0.001, respectively (Figure 2b). By age group, HbA1c levels decreased as follows: in subjects < 65 years of age (n = 65), 8.00% ± 1.59% to 7.29% ± 1.23%, p < 0.001; those 65 to 74 years of age (n = 46), 7.61% ± 1.11% to 7.05% ± 0.99%, p < 0.001; and in those ≥ 75 years of age (n = 51), 7.21% ± 0.87% to 6.75% ± 0.96%, p < 0.001 (Figure 2c).

In each subgroup of baseline HbA1c level [<6.9% (n = 56), 6.9% ≤ baseline HbA1c < 8.4% (n = 89), and 8.4% ≤ baseline HbA1c (n = 43)], the HbA1c levels were decreased at 3 months (−0.19%, -0.43%, and −1.45%, respectively) (Figure 2d). FPG was also decreased after 3 months (n = 84, 145 ± 52 to 129 ± 43 mg/dl, p < 0.001) and 12 months of sitagliptin treatment (n = 65, to 129 ± 42 mg/dl, p = 0.005). The rate of glycemic control achieved (in accordance with the guidelines of the Japanese Diabetes Society) significantly increased (Figure 2e).

### Effects of sitagliptin on blood pressure, lipid profiles and insulin resistance

**Figure 3:**
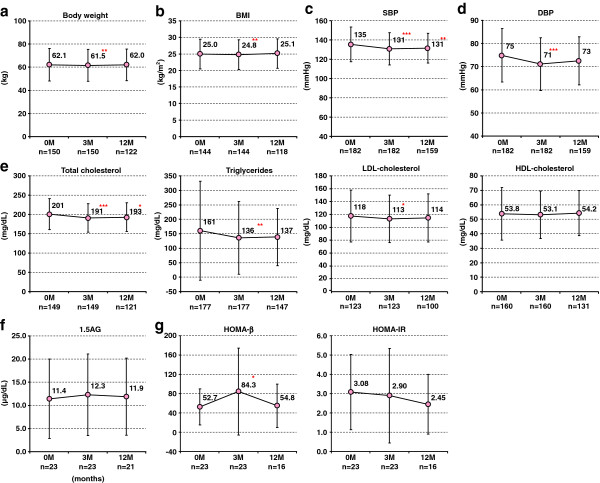
**Serial changes of (a) body weight, (b) BMI, (c) blood pressure, (d) lipid profiles including total cholesterol, triglycerides, LDL-cholesterol and HDL-cholesterol, (e) 1.5 AG, and (f) HOMA-β and –IR.** M, months; SBP, systolic blood pressure; DBP, diastolic blood pressure; LDL, low-density lipoprotein; HDL, high-density lipoprotein; 1.5-AG, 1.5-Anhydro-D-glucitol; HOMA-β, homeostasis model assessment analyses of beta cell function; HOMA-IR, insulin resistance. *p < 0.05, **p < 0.01, ***p < 0.001, vs. baseline by paired t-test.

BW and BMI decreased after 3 months of sitagliptin treatment (BW: 62.1 ± 14.1 to 61.5 ± 13.8 kg, p = 0.003, BMI: 25.0 ± 4.5 to 24.8 ± 4.5 kg/m^2^, p = 0.006). At 12 months, these values had returned to baseline levels (BW: 62.0 ± 13.7 kg, p = 0.800, BMI: 25.1 ± 4.4 kg/m^2^, p = 0.560) (Figure 3a, b). Systolic (SBP) and diastolic blood pressure (DBP) also decreased after 3 months (SBP: 135 ± 18 to 131 ± 17 mmHg, p < 0.001, DBP: 75 ± 12 to 71 ±11 mmHg, p < 0.001) (Figure 3c, d) as did serum levels of TC and TG (TC: 201 ± 40 to 191 ± 37 mg/dl, p < 0.001; TG: 161 ± 171 to 136 ± 126 mg/dl, p = 0.003) (Figure 3e). However, there was no change in the levels of 1.5 AG, HOMA-β, and HOMA-IR observed (Figure 3f, g).

### Effects of sitagliptin on QOL

**Figure 4:**
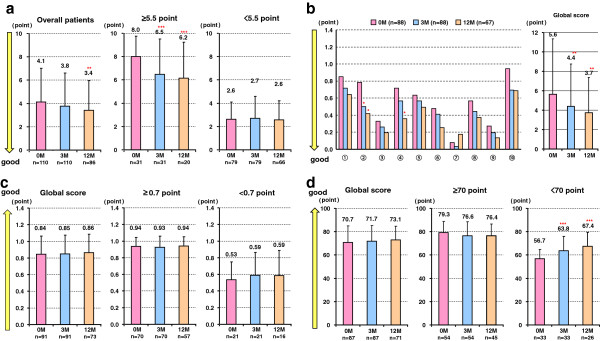
**Effects of sitagliptin on QOL.** (**a**) PSQI the Pittsburgh Sleep Quality Index, (**b**) the Diabetes Symptomatic Score, (**c**) Euro QOL (EQ)-5 Dimensions (EQ-5D), and (**d**) the EQ Visual Analogue Scale (EQ-VAS). *p < 0.05, **p < 0.01, **p < 0.001, vs. baseline by Wilcoxon signed-rank test.

PSQI scores decreased after 12 months of sitagliptin treatment (4.1 ± 2.9 to 3.4 ± 2.5 points, p = 0.007) in all subjects (Figure 4a). In the subgroup of subjects with a PSQI score > 5.5 points, the scores significantly decreased both at 3 months (8.0 ± 1.8 to 6.5 ± 3.0 points, p < 0.001) and 12 months (to 6.2 ± 3.1 points, p < 0.001) after sitagliptin treatment. The Diabetes Symptomatic Scores also decreased at both 3 months (5.6 ± 5.71 to 4.4 ± 4.35, p = 0.004) and 12 months (to 3.7 ± 3.65, p = 0.006) (Figure 4b). Among the 10 diabetes symptomatic questions (Table 2), scores regarding urination (p = 0.013) and paresthesia (p = 0.025) were decreased at 12 months. In contrast, the EQ-5D and EQ-VAS scores did not change significantly (Figure 4c, d).

### Safety

Twenty-three (11%) of the 207 enrolled subjects suffered adverse events (AEs) (Table 3). Two subjects (0.96%) experienced direct sitagliptin-related AEs causing them to discontinue sitagliptin. One subject suffered from gastrointestinal (GI) symptoms, including vomiting, stomachaches, and constipation. The other subject experienced skin eruptions.

**Table 3:** Adverse events

**Event**	**Number**
Death (cause unknown)	1
Worsening of vomiting, stomachache, constipation	1
Loss of consciousness	1
Hypoglycemia	1
Intestinal tuberculosis, pneumonia	1
Stool abnormality	2
Acute myocardial infarction	1
Thrombocytopenia	1
Worsening of heart failure	1
New onset of dyslipidemia	3
Bleeding or ulcer of GI tract	2
Liver dysfunction	4
Elevation of CPK	2
Fever and poor physical health	1
Skin disease including eruption	2
Total number of incidents	24 in 23 subjects

GI, gastric intestinal; CPK, creatinine phosphokinase.

Liver dysfunction is defined as elevated (> 2.5 × the upper limit of normal) alanine aminotransferase or aspartate aminotransferase.

Elevation of CPK is defined as elevated > 2 × the upper limit of normal.

Three subjects (1.15%) developed AEs that were sus-pected to have a causal relationship with sitagliptin. Hypoglycemia and loss of consciousness occurred in two subjects (0.96%). Pneumonia/intestinal tuberculosis and stool abnormality were recognized in one subject.

## Discussion

### Efficacy and safety of sitagliptin

In our study, the HbA1c and FPG levels were reduced at 3 months (HbA1c: 0.59%, FPG: 15.5 mg/dl reduction) and at 12 months (HbA1c: 0.65%, FPG: 20.2 mg/dl reduction) after treatment with sitagliptin at a dose of 25 to 100 mg/day. Our results are similar to those of previous studies reported in the US [13] and Japan [14]. Nathan et al. [15] reported that the expected percentage decrease in HbA1c levels is 1.0% to 2.0% with metformin monotherapy, 1.0% to 2.0% with sulfonylureas (SUs), 0.5% to 1.0% with glinides, 0.5% to 0.8% with α-glucosidase inhibitors (α-GI), 0.5% to 1.4% with thiazolidinediones (TZD) and 0.5% to 0.8% with DPP-4 inhibitors. Monotherapy with metformin or SU exhibits a stronger reduction of HbA1c levels than a DPP-4 inhibitor alone. However, metformin is associated with side effects such as GI symptoms and is contraindicated in patients with renal insufficiency. The major side effects of SUs are hypoglycemia and weight gain. In patients receiving treatment with SUs, the incidence of hypoglycemic episodes has been reported to be 17.6% per year [16]. Side effects appear to be more frequently seen with metformin or SUs than with sitagliptin. The most common side effects of TZD are weight gain and fluid retention along with peripheral edema and an increased risk of congestive heart failure [14,17]. In our study, body weight and BMI decreased and there was no evidence of heart failure during sitagliptin treatment. While metformin, glinides, and α-GIs are required to be taken three times daily, sitagliptin is only taken once daily. Therefore, sitagliptin should be associated with higher adherence compared to metformin, glinides, and α-GIs.

In our study, AEs after sitagliptin treatment were seen in 23 (11%) of the 207 enrolled subjects. In particular, direct sitagliptin-related AEs such as hypoglycemia and loss of consciousness were observed in only two subjects (0.96%). A previous pooled analysis [18] reported that the overall incidence of AEs was similar between sitagliptin (100 mg/day) and other diabetic-comparator agents (except for other DPP-4 inhibitors), including placebos, pioglitazone, metformin, sulfonylureas, sulfonylureas + metformin, and metformin + rosiglitazone (overall side effects: 63.0% vs. 62.8%, hypoglycemia: 3.4% vs. 10.9%). Therefore, incidence of AEs in this study, including hypoglycemia, was lower than that reported in the pooled analysis. This discrepancy appears to be related to differences in dosage. In our study, subjects received doses between 50 and 100 mg/day of sitagliptin with only 24 (11.6%) receiving the highest dose of 100 mg. In the pooled analysis, all subjects received 100 mg/day. In previous studies, sitagliptin did not increase cardiovascular risk in patients with T2DM [19] and sitagliptin reduced postprandial glucose fluctuation and stabilized blood glucose levels effectively in combination with miglitol through continuous glucose monitoring (CGM) [20]. On the other hand, vildagliptin twice a day calmed down the postprandial glucose level as compared to sitagliptin by CGM [21]. The results of this study show that sitagliptin was safe and effective in this population; however, further studies are needed to evaluate the comparison of each DPP-4 inhibitor.

### Effects of DPP-4 inhibitors on blood pressure and lipid profiles

Systolic and diastolic blood pressure decreased after 3 months of treatment with sitagliptin. The active isoforms of GLP-1 include GLP-1(7–36) amide and glycine-extended GLP-1(7–37) [22]. GLP-1(7–36) exhibits vascular actions via GLP-1 receptor signaling [23]. Additionally, GLP-1(9–36), a metabolite of GLP-1 (7–36), has vasodilator effects independent of the GLP-1 receptor in a nitrous oxide/cyclic guanosine monophosphate (cGMP)-dependent manner [23]. DPP-4 inhibitors increase the levels of GLP-1, possibly leading to vasodilatation and blood pressure reduction. In addition, Gutzwiller et al. [24] showed that a pharmacological dose of GLP-1 increases sodium excretion in the proximal renal tubule in obese and insulin-resistant men. Therefore, GLP-1-induced increases in urinary sodium excretion might also contribute to blood pressure reduction after sitagliptin treatment.

In our study, serum levels of TC and TG also decreased after 3 months of treatment with sitagliptin. Qin et al. [25] showed that GLP-1 decreases the intestinal lymph flow and reduces triglyceride absorption and apo B and apo A-IV production in rats. Vildagliptin, another DPP-4 inhibitor, has been shown to reduce the hepatic expression of genes important for cholesterol synthesis, including phosphomevalonate kinase and mevalonate decarboxylase in wild-type mice [26]. Prolonged DPP-4 inhibition modulates the expression of genes important for fatty acid oxidation,including acyl-coenzyme dehydrogenase medium chain and Acyl-CoA synthetase. In addition, DPP-4 inhibitors reduce the levels of hepatic mRNA transcripts for acetyl coenzyme A acyltransferase 1 and carnitine palmitoyltransferase 1, independent of incretin receptor actions [26]. Because these modulations depend on and/or are independent of incretin receptor actions, sitagliptin may have the ability to decrease the levels of TC and TG.

### QOL and diabetes

QOL, whose evaluation is the major goal of our study, is improved after sitagliptin treatment. The sleep quality and PSQI scores decreased after 12 months of treatment with sitagliptin. Particularly, in the subgroup of poor sleepers with PSQI scores above 5.5 points, the scores were significantly reduced not only after 12 months of treatment, but also after 3 months of treatment. Sleep disorders are common in patients with diabetes. Sleep debts decrease carbohydrate tolerance and insulin resistance and increase sympathetic tone, cortisol levels, and nocturnal catecholamine levels [27,28]. Improving sleep disorders with sitagliptin therapy might improve these states, possibly preventing cardiovascular disease in patients with T2DM.

Our own QOL assessment scores for diabetes, the Diabetes Symptomatic Scores, also decreased after sitagliptin treatment. Particularly, the scores regarding urinary frequency and paresthesia of the extremities significantly decreased 12 months after sitagliptin treatment. Urinary frequency, which often appears in T2DM patients, is caused by hyperglycemia-induced polyposia and/or neurogenic bladder. We suppose that polypepsia and polyposia are improved by reductions in FPG after sitagliptin treatment, thereby decreasing the urinary frequency. Paresthesia of the extremities is characterized by striking atrophy and/or loss of myelinated and unmyelinated fibers [29]. Hyperglycemia leads to the development of macrovascular and endoneural microvascular disease in diabetic nerve tissue via several mechanisms, including the polyol pathway. We suppose that both reductions of FPG and vasodilatation, a direct action of DPP-4 inhibitors, leads to improved nerve blood flow and nerve fiber damage in patients with diabetic neuropathy.

The EQ-5D score represents an independent predictor of mortality and future cardiovascular events in patients with T2DM [30]. In our study, however, the EQ-5D scores did not change after treatment with sitagliptin. Because the EQ scores before sitagliptin treatment were as high as 0.84 points, which is close to the cut-off point for a healthy state, they might not change significantly after sitagliptin treatment.

### Limitations/clinical implications

This was a preliminary, single-arm study of a small number of subjects. A large-scale, randomized study conducted over a longer period is needed in the future. However, we found that sitagliptin exerts significant effects, not only on glycemic control, but also on improving QOL, blood pressure, and lipid profiles in subjects with T2DM. Although our results showed efficacy of the drug, we could not precisely evaluate subjects’ adherence to their dosing regimens. As far as we know, there is no reported data on patient adherence to DPP-4 inhibitor treatment regimes. However, adherence to a drug taken once a day is supposed to be higher than conventional drugs taken two or three times a day, which may affect efficacy. In this study, the subjects whose rates of adherence were less than 75% were to be reported as “poor adherence”; all evaluated patients had good adherence to the dosing regimen.

In this study, treatment with sitagliptin achieved adequate reductions in the levels of HbA1c and significant increases in the rate of accomplishment of glycemic control. The use of sitagliptin was shown to be safe and improved the PSQI and Diabetes Symptomatic scores.

## Conclusions

The clinical use of the DPP-4 inhibitor sitagliptin has beneficial effects not only for glucose control, but also for improving blood pressure, lipid profiles, and QOL regarding sleep quality and diabetes symptoms in addition to being safe with a high rate of adherence to treatment.

## Abbreviations

1.5-AG: 1.5-anhydro-D-glucitol; α-GI: α-glucosidase inhibitors; BMI: Body mass index; BW: Body weight; CGM: Continuous glucose monitoring; DPP-4: Dipeptidyl peptidase-4; EQ-5D: Euro QOL-5 dimensions; EQ-VAS: EQ-visual analogue scale; FPG: Fasting plasma glucose; GI: Gastrointestinal; GLP-1: Glucagon-like peptide-1; GIP: Glucose-dependent insulinotropic polypeptide; HDL-C: High density lipoprotein cholesterol; LDL-C: Low density lipoprotein cholesterol; NGSP: National Glycohemoglobin Standardization Program; PSQI: Pittsburgh Sleep Quality Index; QOL: Quality of life; TC: Total cholesterol; TG: Triglycerides; T2DM: Type 2 diabetes mellitus; TZD: Thiazolidinediones; SUs: Sulfonylureas

## Competing interest

The authors declare that they have no conflicts of interest.

## Authors’ contributions

YS, JO, MS, TI, HI, KK, AH, and KN were deeply involved in the conception and design of the study. JO was responsible for the analyses of the data. YS drafted the manuscript. All authors read and approved the final manuscript.

## Authors’ information

**S-DOG investigators:** Saga Challenge Anti-Diabetes Observational Study for Sitagliptin (S-DOG): Shigeki Gondoh, Haruda Yoshio, Minekazu Hashimoto, Hideo Ikeda, Takahiko Imamura, Taketo Iwamoto, Ryota Kaihara, Hideyuki Kamachi, Yoshiyuki Koga, Shigetaka Kuroki, Kazuo Matsunaga, Tadahiro Mizukami, Taizo Minami, Hiroshi Nakanishi, Hirofumi Naito, Masanori Nakao, Masayuki Nakayama, Shinichi Nakayama, Akira Takahashi, Norio Takeda, Otohisa Tajiri, Satoshi Tamesue, Toshifumi Uchida, Yasufumi Uchida, Tetsushi Wakiyama, Tsuneko Yamaguchi, Kenichi Yamamoto and principal investigator Koichi Node.

## References

[B1] Ministry of Health Labour and Welfare of Japan, report of health and nourishment in 2007 2007 Japan

[B2] Gallwitz B Review of sitagliptin phosphate: a novel treatment for type 2 diabetes Vasc Health Risk Manag 2007 3 203 210 10.2147/vhrm.2007.3.2.203 17580730 PMC1994027

[B3] Schmidt WE Siegel EG Creutzfeldt W Glucagon-like peptide-1 but not glucagon-like peptide-2 stimulates insulin release from isolated rat pancreatic islets Diabetologia 1985 28 704 707 10.1007/BF00291980 3905480

[B4] de Heer J Rasmussen C Coy DH Holst JJ Glucagon-like peptide-1, but not glucose-dependent insulinotropic peptide, inhibits glucagon secretion via somatostatin (receptor subtype 2) in the perfused rat pancreas Diabetologia 2008 51 2263 2270 10.1007/s00125-008-1149-y 18795252

[B5] Mentlein R Dipeptidyl-peptidase IV (CD26)–role in the inactivation of regulatory peptides Regul Pept 1999 85 9 24 10.1016/S0167-0115(99)00089-0 10588446

[B6] Brooks R EuroQol: the current state of play Health Policy 1996 37 53 72 10.1016/0168-8510(96)00822-6 10158943

[B7] Lamers L McDonnell J Stalmeier P Krabbe PFM Busschbach JJV The Dutch tariff: results and arguments for an effective design for national EQ-5D valuation studies Health Econ 2006 15 1121 1132 10.1002/hec.1124 16786549

[B8] Dolan P Modeling valuations for EuroQol health states Med Care 1997 35 1095 1108 10.1097/00005650-199711000-00002 9366889

[B9] Brazier J Deverill M Green C Harper R Booth A A review of the use of health status measures in economic evaluation Health Technol Assess 1999 3 1 164 10392311

[B10] Parkin D Rice N Lacoby A Doughty J Use of a visual analogue scale in a daily patient diary: modelling cross-sectional time-series data on health-related quality of life Soc Sci Med 2004 54 351 360 15110425 10.1016/j.socscimed.2003.10.015

[B11] Buysse DJ Reynolds CF III Monk TH Berman SR Kupfer DJ The Pittsburgh Sleep Quality Index: a new instrument for psychiatric practice and research Psychiatry Res 1989 28 193 213 10.1016/0165-1781(89)90047-4 2748771

[B12] Carpenter JS Andrykowski A Psychometric evaluation of the Pittsburgh Sleep Quality Index J Psychosomatic Res 1998 45 5 13 10.1016/S0022-3999(97)00298-5 9720850

[B13] Aschner P Kipnes MS Lunceford JK Sanchez M Mickel C Williams-Herman DE Sitagliptin Study 021 Group Effect of the dipeptidyl peptidase-4 inhibitor sitagliptin as monotherapy on glycemic control in patients with type 2 diabetes Diabetes Care 2006 29 2632 2637 10.2337/dc06-0703 17130196

[B14] Nonaka K Kakikawa T Sato A Okuyama K Fujimoto G Kato N Suzuki H Hirayama Y Ahmed T Davies MJ Efficacy and safety of sitagliptin monotherapy in Japanese patients with type 2 diabetes Diabetes Res Clin Pract 2008 79 291 298 10.1016/j.diabres.2007.08.021 17933414

[B15] Nathan DM Buse JB Davidson MB Ferrannini E Holman RR Sherwin R Zinman B American Diabetes Association, European Association for Study of Diabetes Medical management of hyperglycemia in type 2 diabetes: a consensus algorithm for the initiation and adjustment of therapy: a consensus statement of the American Diabetes Association and the European Association for the Study of Diabetes Diabetes Care 2009 32 193 203 10.2337/dc08-9025 18945920 PMC2606813

[B16] Gangji AS Cukierman T Gerstein HC Goldsmith CH Clase C A systematic review and meta-analysis of hypoglycemia and cardiovascular events: a comparison of glyburide with other secretagogues and with insulin Diabetes Care 2007 30 389 394 10.2337/dc06-1789 17259518

[B17] Singh S Loke YK Furberg CD T**hiazolidinediones and heart failure: a teleo-analysis** Diabetes Care 2007 30 2148 2153 10.2337/dc07-0141 17536074

[B18] Williams-Herman D Round E Swern AS Musser B Davies MJ Stein PP Kaufman KD Amatruda JM Safety and tolerability of sitagliptin in patients with type 2 diabetes: a pooled analysis BMC Endocrine Disorders 2008 8 14 10.1186/1472-6823-8-14 18954434 PMC2605739

[B19] Engel SS Golm GT Shapiro D Davies MJ Kaufman KD Goldstein BJ Cardiovascular safety of sitagliptin in patients with type 2 diabetes mellitus: a pooled analysis Cardiovasc Diabetol 2013 12 3 10.1186/1475-2840-12-3 23286208 PMC3585887

[B20] Kishimoto M Noda M A pilot study of the efficacy of miglitol and sitagliptin for type 2 diabetes with a continuous glucose monitoring system and incretin-related markers Cardiovasc Diabetol 2011 10 115 10.1186/1475-2840-10-115 22189184 PMC3307032

[B21] Sakamoto M Nishimura R Irako T Tsujino D Ando K Utsunomiya K Comparison of vildagliptin twice daily vs. sitagliptin once daily using continuous glucose monitoring (CGM): crossover pilot study (J-VICTORIA study) Cardiovasc Diabetol 2012 11 92 10.1186/1475-2840-11-92 22867630 PMC3471040

[B22] Mojsov S Kopczynski MG Habener JF Both amidated and nonamidated forms of glucagon-like peptide I are synthesized in the rat intestine and the pancreas J Biol Chem 1990 265 8001 8008 1692320

[B23] Ban K Noyan-Ashraf MH Hoefer J Bolz SS Drucker DJ Husain M Cardioprotective and vasodilatory actions of glucagon-like peptide 1 receptor are mediated through both glucagon-like peptide 1 receptor-dependent and independent pathways Circulation 2008 117 2340 2350 10.1161/CIRCULATIONAHA.107.739938 18427132

[B24] Gutzwiller JP Tschopp S Bock A Zehnder CE Huber AR Kreyenbuehl M Gutmann H Drewe J Henzen C Goeke B Glucagon-like peptide 1 induces natriuresis in healthy subjects and in insulin-resistant obese men J Clin Endocrinol Metab 2004 89 3055 3061 10.1210/jc.2003-031403 15181098

[B25] Qin X Shen H Liu M Yang Q Zheng S Sabo M D'Alessio DA Tso P GLP-1 reduces intestinal lymph flow, triglyceride absorption, and apolipoprotein production in rats Am J Physiol Gastrointest Liver Physiol 2005 288 G943 G949 10.1152/ajpgi.00303.2004 15677555

[B26] Flock G Baggio LL Longuet C Drucker DJ Incretin receptors for glucagon-like peptide 1 and glucose-dependent insulinotropic polypeptide are essential for the sustained metabolic actions of vildagliptin in mice Diabetes 2007 56 3006 3013 10.2337/db07-0697 17717280

[B27] Spiegel K Leproult R Van Cauter E Impact of sleep debt on metabolic and endocrine function Lancet 1999 354 1435 1439 10.1016/S0140-6736(99)01376-8 10543671

[B28] Irwin M Thompson J Miller C Gillin JC Ziegler M Effects of sleep and sleep deprivation on catecholamine and interleukin-2 levels in humans: clinical implications J Clin Endocrinol Metab 1999 84 1979 1985 10.1210/jc.84.6.1979 10372697

[B29] Greene DA Sima AA Stevens MJ Feldman EL Lattimer SA Complications: neuropathy, pathogenetic considerations Diabetes Care 1992 15 1902 1925 10.2337/diacare.15.12.1902 1464245

[B30] Clarke PM Hayes AJ Glasziou PG Scott R Simes J Keech AC Using the EQ-5D index score as a predictor of outcomes in patients with type 2 diabetes Med Care 2009 47 61 68 10.1097/MLR.0b013e3181844855 19106732

